# Persistent and Progressive Outer Retina Thinning in Frontotemporal Degeneration

**DOI:** 10.3389/fnins.2019.00298

**Published:** 2019-04-04

**Authors:** Benjamin J. Kim, Murray Grossman, Delu Song, Samantha Saludades, Wei Pan, Sophia Dominguez-Perez, Joshua L. Dunaief, Tomas S. Aleman, Gui-Shuang Ying, David J. Irwin

**Affiliations:** ^1^Department of Ophthalmology, Scheie Eye Institute, Perelman School of Medicine, University of Pennsylvania, Philadelphia, PA, United States; ^2^Department of Neurology, Frontotemporal Lobar Degeneration Center, Perelman School of Medicine, University of Pennsylvania, Philadelphia, PA, United States

**Keywords:** frontotemporal degeneration, optical coherence tomography, retina, tauopathy, progressive supranuclear palsy

## Abstract

**Objective:**

While Alzheimer’s disease is associated with inner retina thinning measured by spectral-domain optical coherence tomography (SD-OCT), our previous cross-sectional study suggested outer retina thinning in frontotemporal degeneration (FTD) patients compared to controls without neurodegenerative disease; we sought to evaluate longitudinal changes of this potential biomarker.

**Methods:**

SD-OCT retinal layer thicknesses were measured at baseline and after 1–2 years. Clinical criteria, genetic analysis, and a cerebrospinal fluid biomarker (total tau: β-amyloid) to exclude likely underlying Alzheimer’s disease pathology were used to define a subgroup of predicted molecular pathology (i.e., tauopathy). Retinal layer thicknesses and rates of change in all FTD patients (*n* = 16 patients, 30 eyes) and the tauopathy subgroup (*n* = 9 patients,16 eyes) were compared to controls (*n* = 30 controls, 47 eyes) using a generalized linear model accounting for inter-eye correlation and adjusting for age, sex, and race. Correlations between retinal layer thicknesses and Mini-Mental State Examinations (MMSE) were assessed.

**Results:**

Compared to controls, returning FTD patients (143 vs. 130 μm, *p* = 0.005) and the tauopathy subgroup (143 vs. 128 μm, *p* = 0.03) had thinner outer retinas but similar inner layer thicknesses. Compared to controls, the outer retina thinning rate was not significant for all FTD patients (*p* = 0.34), but was significant for the tauopathy subgroup (−3.9 vs. 0.4 μm/year, *p* = 0.03). Outer retina thickness change correlated with MMSE change in FTD patients (Spearman rho = 0.60, *p* = 0.02) and the tauopathy subgroup (rho = 0.73, *p* = 0.04).

**Conclusion:**

Our finding of FTD outer retina thinning persists and longitudinally correlates with disease progression. These findings were especially seen in probable tauopathy patients, which showed progressive outer retina thinning.

## Introduction

Frontotemporal degeneration (FTD) syndromes can have clinical presentations that overlap with Alzheimer’s Disease (AD) (Irwin et al., 2015). Up to 30% of clinically diagnosed FTD patients receive a primary neuropathologic diagnosis of AD (Kertesz et al., 2005; Knibb et al., 2006; Irwin et al., 2013). There are predominantly two pathologic FTD subtypes (i.e., frontotemporal lobar degeneration, FTLD): FTLD-Tau, which has inclusions of the microtubule-associated protein tau, and FTLD-TDP, which has TAR DNA-binding protein 43 (TDP-43) inclusions (Irwin et al., 2015). FTD clinical trials are challenged by an inability to determine the causative molecular pathology of patients until autopsy.

Spectral-domain optical coherence tomography (SD-OCT) provides highly reproducible retinal thickness measurements in cognitively impaired patients (Loh et al., 2017). We previously found photoreceptor thinning visualized by SD-OCT in mice with a mutation in *RP1*, a microtubule-associated protein (Song et al., 2014). Since tau is a microtubule-associated protein also expressed in the retina, we hypothesized that tauopathies may have photoreceptor abnormalities detectable by SD-OCT. In a cross-sectional study of FTD patients predominantly composed of probable tauopathy patients, we found that FTD is associated with outer retina (photoreceptor layer) thinning compared to normal controls (Kim et al., 2017). This contrasts with numerous reports of SD-OCT detected inner retina (nerve fiber and ganglion cell layer) thinning associated with AD and confirmed with histopathology (Hinton et al., 1986; Cheung et al., 2015; Coppola et al., 2015; Garcia-Martin et al., 2016; Chan et al., 2018). Furthermore, amyotrophic lateral sclerosis, which has clinical-pathological overlap with FTLD-TDP, is associated with inner retina thinning (Irwin et al., 2015; Volpe et al., 2015).

Longitudinal SD-OCT measurements can provide invaluable data that substantiates cross-sectional data, demonstrates temporal relationships between retinal layers and disease outcomes, and provides causal evidence for the hypothesis that outer retina thinning is associated with progressive tau pathology in FTD. Here, we report longitudinal data for our cohort of deeply phenotyped FTD patients and controls.

## Materials and Methods

### Participants

The recruitment of patients and controls at baseline was described previously (Kim et al., 2017). Briefly, consecutive patients with FTD clinical syndromes [progressive supranuclear palsy (PSP), corticobasal syndrome (CBS), primary progressive aphasia (PPA), and behavioral variant of FTD (bvFTD)] were prospectively enrolled at the Penn Frontotemporal Degeneration Center of the University of Pennsylvania. These patients were all reviewed in a consensus conference and diagnosed according to published clinical criteria (Rascovsky et al., 2011; Irwin et al., 2015; Hoglinger et al., 2017). Neurologists masked to SD-OCT data performed a Mini-Mental State Examination (MMSE) within 12 months of enrollment. Twenty-seven patients were evaluated at baseline.

To determine subgroups, we used the same methodology as the one employed in our previous study (Kim et al., 2017). We used a combination of previously validated cerebrospinal fluid (CSF) (Irwin et al., 2012; Lleo et al., 2018), genetic (Irwin et al., 2015), and clinical criteria (Gorno-Tempini et al., 2011; Irwin et al., 2015; Hoglinger et al., 2017) predictive of underlying pathology to define subgroups (tauopathy, TDP-43, and unknown pathology) of highly predictive pathologic subtypes. First, patients with a total tau:Aβ ratio > 0.34 were considered to have presumed AD and excluded from analyses (Shaw et al., 2009; Irwin et al., 2012, 2013). Next, patients were genotyped according to risk of hereditary disease for pathogenic mutations based on structured pedigree analysis (Wood et al., 2013). This included *MAPT* (OMIM:157140), which is predictive of FTLD-Tau, and the following mutations predictive of FTLD-TDP: progranulin (*GRN*) (OMIM:138945), *C9orf72* (OMIM: 61426), and *TARDBP* p.I383V (OMIM: 605078; p.N39OS). Finally, patients meeting criteria for clinical phenotypes of PSP, non-fluent PPA, and CBS (along with non-AD CSF and absence of GRN mutation) were categorized as FTLD-Tau, as sporadic FTLD-TDP is rare in these clinical phenotypes. Those meeting criteria for the semantic variant of PPA were categorized as FTLD-TDP due to the rarity of sporadic FTLD-Tau with this phenotype (Litvan et al., 1996; Josephs et al., 2006; Irwin et al., 2013; Hoglinger et al., 2017; Spinelli et al., 2017).

At baseline, 44 consecutive healthy controls were prospectively recruited as previously described from the Scheie Eye Institute (Kim et al., 2017). These controls had no history of diabetes or neurodegenerative disease and were originally intended for several SD-OCT studies of different diseases.

From August 2015 to January 2018, we asked patients and controls to return for a single follow-up retinal imaging visit that was approximately 1–2 years after their baseline visit. At this follow-up visit, all participants had another comprehensive, dilated eye examination to diagnose any new ophthalmic disease. Patients also had another neurological exam including a MMSE within 4 months of their follow-up retinal imaging visit. No autopsy data became available for any patient with a follow-up visit.

The University of Pennsylvania Institutional Review Board approved this study, and all participants (or caregivers when appropriate) provided written informed consent in accordance with the Declaration of Helsinki.

### SD-OCT Protocol and Image Analysis

**FIGURE 1:**
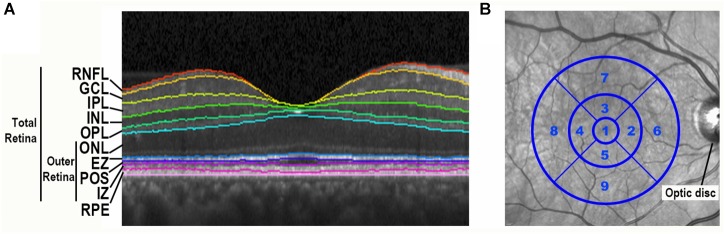
Spectral-domain optical coherence tomography retinal image segmentation and ETDRS grid centered on the fovea. **(A)** Segmentation of a spectral-domain optical coherence tomography (SD-OCT) retina image by the Iowa Reference Algorithm. A portion of a SD-OCT image taken through the fovea is shown with retinal layers labeled. RNFL, retinal nerve fiber layer; GCL, ganglion cell layer; IPL, inner plexiform layer; INL, inner nuclear layer; OPL, outer plexiform layer; ONL, outer nuclear layer; EZ, ellipsoid zone; POS, photoreceptor outer segments; IZ, interdigitation zone; RPE, retinal pigment epithelium. **(B)** The standard Early Treatment Diabetic Retinopathy Study (ETDRS) grid is centered on the fovea of an infrared fundus image.

At the follow-up visit, all participants completed an SD-OCT imaging protocol using the same methodology as the one employed in our baseline study (Kim et al., 2017). All participants underwent SD-OCT imaging with the Heidelberg Spectralis (Heidelberg Engineering, Carlsbad, CA, United States) with a standard macular volume scan protocol of 20° images, 25 high-resolution raster scans, and automated real time averaging of 25. Images met OSCAR-IB quality control criteria that relate to macular volume scans (Tewarie et al., 2012). An analyst masked to clinical information segmented individual retinal layers using the well-validated Iowa Reference Algorithm (IRA) (v3.6), and algorithm segmentation errors were manually corrected (Li et al., 2006; Garvin et al., 2009; Abramoff et al., 2010). This algorithm segmented 11 optical interfaces (10 layers) and provided thickness readings for the 9 regions of the standard Early Treatment of Diabetic Retinopathy Study (ETDRS) grid centered at the fovea. Total retina thickness (neurosensory retina) was defined as the distance from the retinal nerve fiber layer’s inner boundary, including the internal limiting membrane, to the interdigitation zone’s outer boundary. To focus on photoreceptor layers, the outer retina thickness was defined as the distance from the outer nuclear layer’s (ONL) inner boundary to the interdigitation zone’s outer boundary (Figure 1A). With this definition, the outer retina thickness does not include the outer plexiform layer (OPL), as the OPL thickness is partially composed of horizontal and bipolar cell dendrites. Remaining consistent with our baseline study, analyses of retinal layers focused on the average of the central 5 regions of the fovea-centered ETDRS grid, which is an area with a 3 mm diameter (Figure 1B).

### Statistical Analyses

**FIGURE 2:**
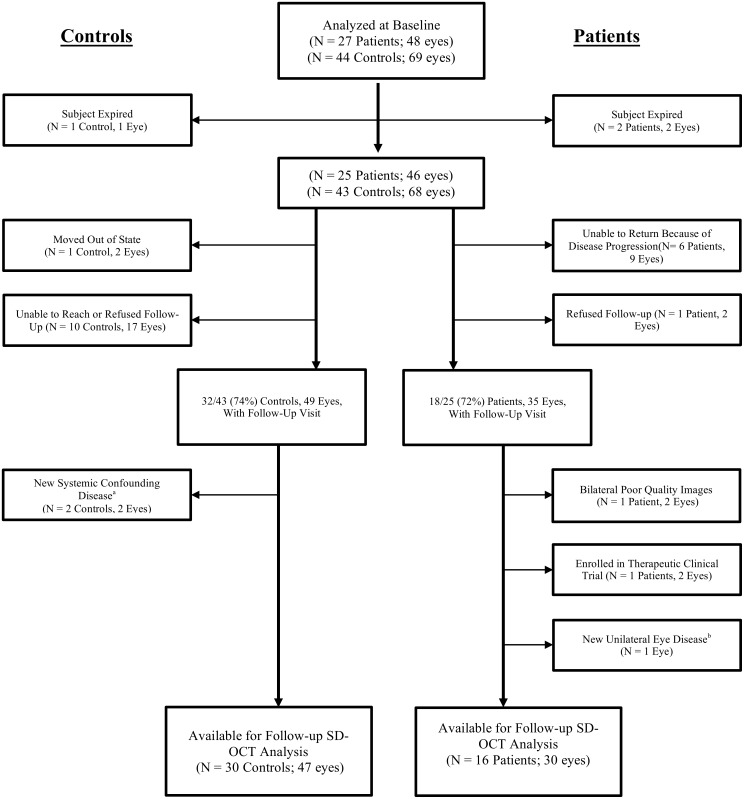
Flow diagram of excluded subjects and eyes prior to analysis. Every patient was diagnosed with a frontotemporal degeneration clinical syndrome. SD-OCT, spectral domain optical coherence tomography. ^a^One control received a new diagnosis of Parkinson’s disease. One control developed severe systemic hypertension with evidence of new hypertensive retinopathy in the study eye. ^b^One patient developed mild macroaneurysm of a retinal vessel at the macula in one eye.

As pre-specified, we excluded participants (or eyes) with eye diseases that may affect the retinal thickness including macular disease, retinal vascular disease, diabetic or hypertensive retinopathy, glaucoma or optic nerve disease, significant ocular media opacity, high refractive error (±6.00 diopters spherical equivalent), intraocular surgery within 90 days, or poor image quality (Figure 2).

We calculated the annual rate (microns per year) of retinal layer thickness change as: [(follow-up visit thickness – baseline thickness)/(months of follow-up)] × 12. We compared each of the retinal layer thicknesses measured at the follow-up time point and its annual rate of change from baseline between all patients versus controls and between each of the subgroups (tauopathy, TDP-43, unknown pathology) versus controls using generalized linear models (Liang and Zeger, 1986). These models were performed with and without adjustment for participant demographics, as age, sex, and race are known to affect retinal layer thicknesses in normal subjects (Kashani et al., 2010; Girkin et al., 2011; Demirkaya et al., 2013). The statistical adjustment for age, sex, and race is performed by including these items as covariates in the multivariable regression models for comparing retinal thickness between FTD and controls. For participants with two study eyes eligible for this study, inter-eye correlations were accounted for by using the generalized estimating equations (GEE) (Liang and Zeger, 1986). The GEE was initially developed to analyze correlated data from longitudinal repeated measures; it has been commonly applied to analyze correlated eye data, accounting for the inter-eye correlation when using the eye as the unit of analysis (Ying et al., 2017).

For all patients and the probable tauopathy subgroup, we calculated the Spearman correlation of MMSE with outer retina thickness, ONL, and ellipsoid zone (EZ) thickness. For the Spearman correlation, the average thickness of 2 eyes was used for participants with data from 2 study eyes. All statistical analyses were performed with SAS v9.4 (SAS Institute Inc., Cary, NC, United States). Two-sided *p* < 0.05 was considered statistically significant.

### Data Availability

Anonymized data for this study will be shared by request from any qualified investigator.

## Results

### Demographics

The study enrolled 27 FTD patients and 44 controls without neurodegenerative disease. After the baseline visit, two FTD patients expired and therefore were unavailable for a follow-up visit. Eighteen of the remaining 25 (72%) patients completed a follow-up visit at a mean of 15.6 months after the baseline visit (Table 1). Of 44 controls, 1 participant expired after baseline, and 32 of the remaining 43 (74%) controls completed a follow-up visit at a mean of 14.5 months after the baseline visit. Figure 2 details the reasons for missed follow-up and exclusion from statistical analyses. Ultimately, 16 FTD patients (30 eyes) and 30 controls (47 eyes) were included in longitudinal data analyses. As expected from the baseline demographics, compared to controls, the FTD patients had a similar percentage of male participants but had a greater mean age and a higher percentage of Caucasian race (Table 1). All but one of the patients had CSF biomarker analysis to exclude AD. This patient met clinical criteria for PSP, which neuropathologically is highly specific for a tauopathy (Litvan et al., 1996; Josephs et al., 2006; Hoglinger et al., 2017).

**Table 1:** Demographic characteristics of FTD patients and controls.

		Controls (*N* = 30)	All FTD patients (*N* = 16)	*p*-value^a^	Probable tauopathy subgroup (*N* = 9)	*p*-value^b^
Months of follow-up						
	Mean (*SD*)	14.5 (3.2)	15.6 (4.4)	0.31	15.2 (4.2)	0.56
	Minimum, Maximum	12, 25	11, 24		11, 24	
Age at baseline (years)						
	Mean (*SD*)	53.1 (11.3)	65.6 (8.2)	<0.001	65.8 (6.7)	<0.001
	Minimum, Maximum	26, 77	53, 87		53, 76	
Sex, *n* (%)						
	Male	9 (30.0)	6 (37.5)	0.53	2 (22.2)	1.00
	Female	21 (70.0	10 (62.5)		7 (77.8)	
Race, *n* (%)						
	Caucasian	16 (53.3)	13 (81.3)	<0.001	7 (77.8)	0.01
	African–American	12 (40.0)	0 (0.0)		0 (0.0)	
	Other/unknown	2 (6.7)	3 (18.8)		2 (22.2)	

*FTD, frontotemporal degeneration; SD, standard deviation. ^a^p-value for comparison of controls and patients. ^b^p-value for comparison of controls and probable tauopathy subgroup.*

Nine patients (16 eyes) met criteria for the probable tauopathy subgroup and their demographics are shown in Table 1. This subgroup included patients with the following clinical diagnoses: 4 PSP, 3 CBS, 1 non-fluent PPA, and one patient with behavioral variant of FTD with features of the semantic variant of PPA that had a *MAPT* E10+16 C > T mutation.

### Comparison of Retinal Thicknesses at Follow-Up Between Patients and Controls

First, we compared the retinal layer thicknesses between all FTD patients and controls cross-sectionally at the follow-up visit time-point only. For the outer retina thickness, we used our pre-specified definition (see section “Materials and Methods” and Figure 1A) which includes all retinal photoreceptor layers but does not include the outer plexiform layer (OPL) as the OPL thickness is composed, in part, of horizontal and bipolar cell dendrites. Compared to controls, FTD patients had a thinner ONL, EZ, and outer retina thickness in both univariate analysis and multivariate analysis adjusted for age, sex, and race (all *p* < 0.05, Table 2). Observed outer retina thickness values at baseline and follow-up for each eye are shown in Table 3 for FTD patients and Table 4 for controls. The OPL of FTD patients was thicker than that of controls in univariate analysis (*p* = 0.01), but this was not significant in multivariate analysis (*p* = 0.053, Table 2).

**Table 2:** Comparisons of retinal layer thicknesses (microns) between all patients and normal controls at follow-up visit.

	Unadjusted analysis			Adjusted analysis^c^		
Retinal layer^a^	Controls (*N* = 30, 47 eyes) Mean (*SE*)	Patients (*N* = 16, 30 eyes) Mean (*SE*)	*p*-value^b^	Controls (*N* = 30, 47 eyes) Mean (*SE*)	Patients (*N* = 16, 30 eyes) Mean (*SE*)	*p*-value^b^
Total retina	304 (2.3)	301 (3.1)	0.47	306 (3.1)	299 (4.6)	0.27
Outer retina	142 (1.4)	133 (2.1)	**0.008**	143 (1.6)	130 (2.9)	**0.005**
RNFL	23.5 (0.4)	24.3 (0.6)	0.35	23.8 (0.5)	23.9 (0.8)	0.87
GCL	39.6 (1.1)	39.5 (1.1)	0.96	38.2 (1.6)	41.4 (1.7)	0.28
IPL	38.9 (0.9)	39.6 (1.1)	0.68	39.6 (1.3)	39.0 (1.4)	0.89
INL	35.7 (0.5)	35.4 (0.8)	0.78	36.1 (0.6)	35.0 (1.0)	0.47
OPL	24.5 (0.6)	29.2 (1.2)	**0.01**	24.7 (0.8)	28.7 (1.4)	0.053
ONL	96.1 (1.2)	90.1 (1.8)	**0.04**	98.6 (1.5)	86.3 (2.3)	**0.003**
EZ	15.1 (0.1)	14.2 (0.1)	<**0.001**	15.1 (0.2)	14.3 (0.2)	**0.007**
POS	11.8 (0.4)	10.7 (0.5)	0.22	11.4 (0.6)	11.4 (0.8)	0.99
IZ	18.9 (0.5)	17.6 (0.5)	0.17	18.3 (0.6)	18.5 (0.8)	0.88
RPE	18.2 (0.5)	19.0 (0.7)	0.43	18.7 (0.7)	18.2 (0.9)	0.70

*SE, standard error; RNFL, retinal nerve fiber layer; GCL, ganglion cell layer; IPL, inner plexiform layer; INL, inner nuclear layer; OPL, outer plexiform layer; ONL, outer nuclear layer; EZ, ellipsoid zone; POS, photoreceptor outer segments; IZ, interdigitation zone; RPE, retinal pigment epithelium. ^a^Spectral domain optical coherence tomography (SD-OCT) parameters (in microns) are the average of five central subfields (central and 4 parafoveal subfields) of the Early Treatment Diabetic Retinopathy Study (ETDRS) grid centered at the foveola. Total retina: distance from RNFL to IZ; Outer Retina: distance from ONL to IZ. ^b^Inter-eye correlation of SD-OCT measurements was accounted for using generalized estimating equations (GEE); significant values (p < 0.05) in bold letters. ^c^Adjusted for age, sex, and race.*

**Table 3:** Outer retina thickness measurements (microns) at baseline and follow-up for frontotemporal degeneration patients (*N* = 16 patients, 30 eyes).

Subject number	Subgroup	Eye	Baseline outer retina thickness	Follow-up outer retina thickness	Change in outer retina thickness	Length of follow-up (months)
1	Probable tauopathy	OS	144.7	142.9	−1.8	12
2	Probable tauopathy	OD	108.8	109.4	0.6	11
		OS	120.8	118.0	−2.8	11
3	Probable tauopathy	OD	143.2	145.1	1.9	13
		OS	143.9	147.7	3.9	13
4	Probable tauopathy	OD	130.2	127.0	−3.2	19
		OS	132.9	123.8	−9.1	19
5	Probable tauopathy	OD	129.1	115.3	−13.7	18
		OS	130.5	127.2	−3.3	18
6	Probable tauopathy	OD	140.6	136.6	−4.0	14
		OS	143.7	142.5	−1.2	14
7	Probable tauopathy	OD	140.7	127.4	−13.3	12
		OS	124.1	125.3	1.2	12
8	Probable tauopathy	OS	150.5	151.7	1.2	12
9	Probable tauopathy	OD	141.8	122.0	−19.8	24
		OS	136.8	121.2	−15.6	24
10	Probable TDP	OD	123.4	146.5	23.1	12
		OS	117.1	124.3	7.2	12
11	Probable TDP	OD	124.5	136.6	12.1	24
		OS	142.1	140.5	−1.6	24
12	Unknown pathology	OD	151.2	152.6	1.4	12
		OS	137.0	149.4	12.4	12
13	Unknown pathology	OD	122.8	133.0	10.3	21
		OS	137.8	118.4	−19.4	21
14	Unknown pathology	OD	128.5	125.3	−3.2	13
		OS	142.5	130.2	−12.2	13
15	Unknown pathology	OD	137.8	130.1	−7.7	18
		OS	140.5	141.5	1.0	18
16	Unknown pathology	OD	133.4	130.7	−2.8	12
		OS	134.7	136.3	1.5	12

*TDP, TAR DNA binding protein.*

**Table 4:** Outer retina thickness measurements (microns) at baseline and follow-up for normal controls (*N* = 30 subjects, 47 eyes).

Subject number	Eye	Baseline outer retina thickness	Follow-up outer retina thickness	Change in outer retina thickness	Length of follow-up (months)
1	OD	145.6	144.9	−0.7	13
	OS	144.5	146.0	1.6	13
2	OD	120.9	118.5	−2.3	13
	OS	121.8	120.0	−1.8	13
3	OS	149.9	149.6	−0.3	13
4	OD	137.8	138.9	1.1	24
	OS	145.5	147.3	1.8	24
5	OD	136.7	144.0	7.2	13
6	OS	139.2	139.3	0.1	13
7	OD	147.1	147.3	0.2	13
	OS	149.1	150.3	1.1	13
8	OD	143.5	139.8	−3.8	13
	OS	140.8	139.3	−1.4	13
9	OD	138.2	128.2	−10.0	12
	OS	135.0	138.9	4.0	12
10	OS	147.0	152.9	6.0	12
11	OS	139.0	136.2	−2.7	13
12	OD	150.5	156.2	5.7	13
	OS	157.3	161.0	3.7	13
13	OD	145.7	147.3	1.7	25
14	OD	151.6	149.0	−2.7	13
	OS	155.1	151.1	−4.0	13
15	OD	137.8	137.1	−0.7	13
	OS	135.6	133.6	−2.1	13
16	OS	130.4	140.3	9.9	13
17	OD	147.2	145.5	−1.7	13
	OS	135.9	133.6	−2.4	13
18	OD	152.0	151.2	−0.8	13
19	OD	135.6	141.4	5.8	13
	OS	132.0	128.8	−3.2	13
20	OS	176.8	166.7	−10.0	16
21	OD	145.4	141.2	−4.2	20
	OS	135.4	139.8	4.4	20
22	OD	138.5	134.7	−3.8	14
	OS	141.3	137.2	−4.1	14
23	OD	138.8	134.0	−4.8	17
	OS	140.2	144.1	3.8	17
24	OD	149.0	142.1	−6.9	18
	OS	142.2	140.3	−1.9	18
25	OD	122.2	124.2	1.9	12
26	OD	146.1	139.2	−7.0	13
27	OD	148.4	149.0	0.6	13
28	OD	132.2	140.2	8.0	14
	OS	138.6	138.0	−0.6	14
29	OD	142.6	144.9	2.3	14
	OS	145.7	144.3	−1.4	14
30	OS	132.3	154.6	22.3	17

Next, we compared the retinal layer thicknesses at the follow-up visit between only the probable tauopathy subgroup and controls. The tauopathy subgroup had a significantly thinner EZ and outer retina thickness than controls, even after adjusting by age, sex, and race (all *p* < 0.05, Table 5). Tauopathy patients also had a significantly thinner total retina thickness in the univariate analysis (*p* = 0.04), but this difference was not significant after adjusting for age, sex, and race (*p* = 0.07, Table 5). There was non-significant thinning of the ONL in the univariate analysis (*p* = 0.11) and this became significant (*p* = 0.02) in the multivariate analysis.

**Table 5:** Comparisons of retinal layer thicknesses (microns) between probable tauopathy subgroup patients and normal controls at follow-up visit.

	Unadjusted analysis			Adjusted analysis^c^		
Retinal layer^a^	Controls (*N* = 30, 47 eyes) Mean (*SE*)	Probable tauopathy patients (*N* = 9, 16 eyes) Mean (*SE*)	*p*-value^b^	Controls (*N* = 30, 47 eyes) Mean (*SE*)	Probable tauopathy patients (*N* = 9, 16 eyes) Mean (*SE*)	*p*-value^b^
Total retina	304 (2.3)	292 (2.9)	**0.04**	305 (2.9)	291 (4.9)	0.07
Outer retina	142 (1.4)	130 (3.2)	**0.03**	143 (1.6)	128 (4.4)	**0.03**
RNFL	23.5 (0.4)	23.5 (0.9)	0.96	23.5 (0.5)	23.4 (1.3)	0.92
GCL	39.6 (1.1)	37.7 (1.1)	0.33	38.9 (1.4)	39.7 (2.1)	0.79
IPL	38.9 (0.9)	37.5 (1.4)	0.49	39.1 (1.1)	36.9 (1.9)	0.40
INL	35.7 (0.5)	34.0 (0.7)	0.14	35.8 (0.6)	33.8 (0.8)	0.11
OPL	24.5 (0.6)	29.5 (1.9)	0.07	24.9 (0.8)	28.4 (2.2)	0.19
ONL	96.1 (1.2)	89.1 (2.5)	0.11	97.6 (1.4)	85.2 (3.7)	**0.02**
EZ	15.1 (0.1)	14.0 (0.1)	**0.002**	15.0 (0.2)	14.3 (0.2)	**0.02**
POS	11.8 (0.4)	10.1 (0.5)	0.09	11.6 (0.6)	10.7 (1.0)	0.49
IZ	18.9 (0.5)	17.0 (0.7)	0.13	18.4 (0.6)	18.2 (1.1)	0.86
RPE	18.2 (0.5)	20.1 (0.8)	0.17	18.5 (0.6)	19.1 (1.1)	0.67

*SE, standard error; RNFL, retinal nerve fiber layer; GCL, ganglion cell layer; IPL, inner plexiform layer; INL, inner nuclear layer; OPL, outer plexiform layer; ONL, outer nuclear layer; EZ, ellipsoid zone; POS, photoreceptor outer segments; IZ, interdigitation zone; RPE, retinal pigment epithelium. ^a^Spectral domain optical coherence tomography (SD-OCT) parameters (in microns) are the average of five central subfields (central and 4 parafoveal subfields) of the Early Treatment Diabetic Retinopathy Study (ETDRS) grid centered at the foveola. Total retina: distance from RNFL to IZ; Outer Retina: distance from ONL to IZ. ^b^Inter-eye correlation of SD-OCT measurements was accounted for using generalized estimating equations (GEE); significant values (p < 0.05) in bold letters. ^c^Adjusted for age, sex, and race.*

### Comparisons of the Rate of Retinal Layer Thickness Change Between Patients and Controls

We compared the annual rate of change for the retinal layer thickness between all FTD patients and controls. The FTD patients did not show a significant difference in the rate of change of retinal layer thicknesses as compared to controls (Table 6). After adjusting for age, sex, and race, the FTD patients demonstrated an inner nuclear layer rate of thinning that was of borderline significance compared to controls (−0.5 vs. +0.2 μm/year, *p* = 0.046). There was no significant difference in the rate of outer retina change (*p* = 0.34, Table 6).

**Table 6:** Comparisons of rate (microns per year) of retinal layer thickness change between all patients and controls.

	Unadjusted analysis			Adjusted analysis^c^		
Retinal layer^a^	Controls (*N* = 30, 47 eyes) Mean (*SE*)	Patients (*N* = 16, 30 eyes) Mean (*SE*)	*p*-value^b^	Controls (*N* = 30, 47 eyes) Mean (*SE*)	Patients (*N* = 16, 30 eyes) Mean (*SE*)	*p*-value^b^
Total retina	0.5 (0.6)	0.1 (0.9)	0.81	0.9 (0.8)	−0.2 (1.3)	0.60
Outer retina	0.1 (0.7)	−0.9 (1.3)	0.52	0.7 (0.8)	−1.5 (1.6)	0.34
RNFL	0.3 (0.3)	0.9 (0.4)	0.31	0.3 (0.4)	1.0 (0.6)	0.36
GCL	−1.0 (0.5)	−0.9 (1.1)	0.95	−1.3 (0.8)	−0.4 (1.3)	0.61
IPL	1.1 (0.5)	0.1 (1.0)	0.38	1.6 (0.8)	−0.4 (1.2)	0.26
INL	0.0 (0.2)	−0.2 (0.2)	0.42	0.2 (0.2)	−0.5 (0.2)	**0.046**
OPL	−0.2 (0.4)	1.1 (0.8)	0.22	−0.5 (0.5)	1.5 (0.9)	0.09
ONL	−0.1 (0.4)	−1.0 (0.9)	0.45	0.3 (0.6)	−1.5 (1.1)	0.23
EZ	−0.1 (0.1)	−0.3 (0.1)	0.11	−0.1 (0.1)	−0.3 (0.1)	0.23
POS	0.1 (0.1)	0.1 (0.2)	0.91	0.1 (0.2)	0.2 (0.3)	0.73
IZ	0.2 (0.3)	0.2 (0.4)	0.94	0.3 (0.3)	0.1 (0.4)	0.82
RPE	−0.1 (0.3)	−0.1 (0.5)	0.93	−0.3 (0.3)	0.1 (0.5)	0.69

*SE, standard error; RNFL, retinal nerve fiber layer; GCL, ganglion cell layer; IPL, inner plexiform layer; INL, inner nuclear layer; OPL, outer plexiform layer; ONL, outer nuclear layer; EZ, ellipsoid zone; POS, photoreceptor outer segments; IZ, interdigitation zone; RPE, retinal pigment epithelium. ^a^Spectral domain optical coherence tomography (SD-OCT) parameters (in microns) are the average of five central subfields (central and 4 parafoveal subfields) of the Early Treatment Diabetic Retinopathy Study (ETDRS) grid centered at the foveola. Total retina: distance from RNFL to IZ; Outer Retina: distance from ONL to IZ. ^b^Inter-eye correlation of SD-OCT measurements was accounted for using generalized estimating equations (GEE); significant values (p < 0.05) in bold letters. ^c^Adjusted for age, sex, and race.*

**FIGURE 3:**
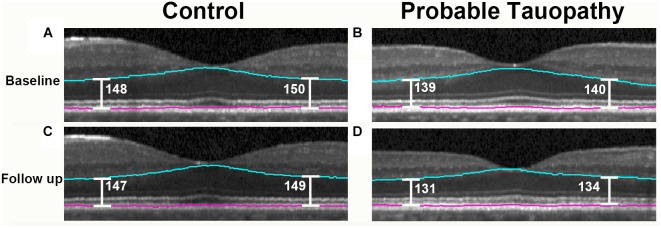
Probable tauopathy patient with progressive outer retina thinning compared to a control. Representative spectral domain optical coherence tomography (SD-OCT) images centered on the fovea are shown. A 63 year-old Caucasian male control at baseline and 13 months later are shown in **(A,C)**. These images are compared with a 69 year-old Caucasian male probable tauopathy patient at baseline and 12 months later as shown in **(B,D)**. The Iowa Reference Algorithm segmentation lines outlining the outer retina are shown in blue and pink. To show outer retina thickness measurements in these images, point measurements were made nasal and temporal from the foveal center using the caliper function of the Heidelberg Spectralis (Heidelberg Engineering, Carlsbad, CA, United States) and labeled in white.

When evaluating only the probable tauopathy subgroup compared to controls, we found a significant rate of thinning of the outer retina (*p* = 0.04) and a significant rate of thickening of the OPL (*p* = 0.02) in univariate analysis (Table 7). After adjusting for age, sex, and race, the probable tauopathy subgroup showed a significant rate of thinning of the ONL (*p* = 0.04), inner nuclear layer (INL, *p* = 0.02), and outer retina (*p* = 0.03) compared to controls (Table 7). The rate of OPL thickening remained significant (*p* = 0.008). Figure 3 shows representative images of progressive outer retina thinning in a probable tauopathy patient compared to a control of similar demographics.

**Table 7:** Comparisons of rate (microns per year) of retinal layer thickness change between probable tauopathy subgroup patients and controls.

	Unadjusted analysis			Adjusted analysis^c^		
Retinal layer^a^	Controls (*N* = 30, 47 eyes) Mean (*SE*)	Patients (*N* = 9, 16 eyes) Mean (*SE*)	*p*-value^b^	Controls (*N* = 30, 47 eyes) Mean (*SE*)	Patients (*N* = 9, 16 eyes) Mean (*SE*)	*p*-value^b^
Total retina	0.5 (0.6)	−0.8 (0.7)	0.29	0.8 (0.7)	−1.8 (1.0)	0.09
Outer retina	0.1 (0.7)	−3.2 (1.2)	**0.04**	0.4 (0.7)	−3.9 (1.3)	**0.03**
RNFL	0.3 (0.3)	0.8 (0.4)	0.32	0.3 (0.4)	0.8 (0.6)	0.52
GCL	−1.0 (0.5)	0.8 (1.1)	0.24	−1.2 (0.7)	1.4 (1.8)	0.24
IPL	1.1 (0.5)	−1.4 (1.0)	0.08	1.5 (0.7)	−2.5 (1.6)	0.06
INL	0.0 (0.2)	−0.5 (0.3)	0.11	0.2 (0.2)	−0.9 (0.3)	**0.02**
OPL	−0.2 (0.4)	2.7 (0.8)	**0.02**	−0.3 (0.4)	3.3 (0.9)	**0.008**
ONL	−0.1 (0.4)	−2.2 (0.8)	0.08	0.1 (0.5)	−2.9 (1.1)	**0.04**
EZ	−0.1 (0.1)	−0.4 (0.1)	0.09	−0.1 (0.1)	−0.4 (0.2)	0.22
POS	0.1 (0.1)	0.1 (0.3)	0.90	0.1 (0.2)	0.0 (0.3)	0.84
IZ	0.2 (0.3)	−0.6 (0.4)	0.11	0.2 (0.3)	−0.7 (0.5)	0.14
RPE	−0.1 (0.3)	0.9 (0.6)	0.12	−0.2 (0.3)	1.0 (0.7)	0.14

*SE, standard error; RNFL, retinal nerve fiber layer; GCL, ganglion cell layer; IPL, inner plexiform layer; INL, inner nuclear layer; OPL, outer plexiform layer; ONL, outer nuclear layer; EZ, ellipsoid zone; POS, photoreceptor outer segments; IZ, interdigitation zone; RPE, retinal pigment epithelium. ^a^Spectral domain optical coherence tomography (SD-OCT) parameters (in microns) are the average of five central subfields (central and 4 parafoveal subfields) of the Early Treatment Diabetic Retinopathy Study (ETDRS) grid centered at the foveola. Total retina: distance from RNFL to IZ; Outer Retina: distance from ONL to IZ. ^b^Inter-eye correlation of SD-OCT measurements was accounted for using generalized estimating equations (GEE); significant values (p < 0.05) in bold letters. ^c^Adjusted for age, sex, and race.*

### Retinal Thickness Correlations With the Change in Disease Severity

Using the retinal layer thicknesses (ONL, EZ, and outer retina) that we found to be different from controls, we evaluated the correlation of these SD-OCT retinal thicknesses and MMSE. Among all FTD patients, the baseline measurements of outer retina, ONL, and EZ thicknesses were not significantly correlated with the change of MMSE (all *p* ≥ 0.60, Table 8). However, the SD-OCT follow-up measurements of the ONL (rho = 0.54, *p* = 0.04) and outer retina (rho = 0.56, *p* = 0.03) were positively correlated with the follow-up MMSE. The change of thickness of the ONL (rho = 0.69, *p* = 0.005) and outer retina (rho = 0.79, *p* < 0.001) were also positively correlated with the follow-up MMSE. Additionally, the change of thickness of the outer retina were positively correlated with the change of MMSE (rho = 0.60, *p* = 0.02, Table 8).

**Table 8:** Spearman correlation between retinal layers and Mini-Mental State Examination for all FTD patients and probable tauopathy subgroup.

	Baseline SD-OCT with change of MMSE		Follow-up SD-OCT with follow-up MMSE		Change of SD-OCT with follow-up MMSE		Change of SD-OCT with change of MMSE		
Retinal layer thickness^a^	rho^b^	*p*-value^c^	rho^d^	*p*-value^c^	rho^d^	*p*-value^c^	rho^d^	*p*-value^c^	Patient group
Outer retina	−0.12	0.65	0.56	**0.03**	0.79	**<0.001**	0.60	**0.02**	FTD patients
Outer nuclear layer	0.02	0.93	0.54	**0.04**	0.69	**0.005**	0.46	0.09	
EZ	0.14	0.60	0.19	0.49	0.13	0.65	0.19	0.49	
									
Outer retina	0.43	0.25	0.56	0.15	0.75	**0.03**	0.73	**0.04**	Probable tauopathy subgroup
Outer nuclear layer	0.49	0.18	0.51	0.19	0.51	0.19	0.55	0.16	
EZ	0.38	0.31	0.08	0.84	−0.14	0.73	−0.04	0.93	

*Significant values (p < 0.05) in bold letters. SD-OCT, spectral domain optical coherence tomography; MMSE, mini-mental state examination; EZ, ellipsoid zone; FTD, frontotemporal degeneration. ^a^Correlation was determined with the average of the 5 central subfields of the Early Treatment Diabetic Retinopathy Study grid. ^b^For FTD patients: N = 16; median number of months between baseline and follow-up MMSE = 17 (interquartile range, 12–24 months); median baseline MMSE = 28 (interquartile range, 26–29); median follow-up MMSE = 26 (interquartile range, 23–28). For probable tauopathy subgroup: N = 9; median number of months between baseline and follow-up MMSE = 15 (interquartile range, 12–27 months); median baseline MMSE = 26 (interquartile range, 24–29); median follow-up MMSE = 25 (interquartile range, 20–28). ^c^For subjects with 2 study eyes, the average of the 2 eyes was correlated. ^d^For FTD patients: N = 15; median number of months between baseline and follow-up MMSE = 17 (interquartile range, 12–25 months); median baseline MMSE = 28 (interquartile range, 26–29); median follow-up MMSE = 26 (interquartile range, 24–28). For probable tauopathy subgroup: N = 8; median number of months between baseline and follow-up MMSE = 19 (interquartile range, 13–27 months); median baseline MMSE = 27 (interquartile range, 24–29); median follow-up MMSE = 26 (interquartile range, 22–28).*

Among only the probable tauopathy subgroup, the change of thickness of the outer retina was positively correlated with the follow-up MMSE (rho = 0.75, *p* = 0.03). Furthermore, the change of the outer retina thickness was positively correlated with the change of the MMSE (rho = 0.73, *p* = 0.04, Table 8).

### Analysis of TDP-43 and Unknown Pathology Subgroups

There were two probable TDP-43 patients (four eyes), including one patient with a hexanucleotide expansion in *C9orf72*. Compared to controls, the probable TDP subgroup had no significant findings for the thickness of any of the retinal layers in unadjusted analysis and an adjusted analysis (data not shown). Similarly, compared to controls, the probable TDP subgroup had no significant findings for the rate of thickness change of any of the retinal layers in unadjusted and an adjusted analysis (data not shown).

Five behavioral variant of FTD patients (10 eyes) were categorized as unknown molecular pathology due to the poor predictive value for molecular pathology of this clinical FTD syndrome (Irwin et al., 2015). Compared to controls, the unknown pathology subgroup had no significant findings for the thickness of any of the retinal layers in unadjusted analysis and an adjusted analysis (data not shown). Similarly, compared to controls, the unknown pathology subgroup had no significant findings for the rate of thickness change of any of the retinal layers in unadjusted and an adjusted analysis (data not shown).

## Discussion

Spectral-domain optical coherence tomography imaging is increasingly investigated as a biomarker for neurodegenerative conditions. Before using this potential biomarker in clinical care or clinical trials, longitudinal data is critically important to understand the rate of retinal layer change and how retinal layer thicknesses temporally relate to disease subgroups and outcomes. Longitudinal SD-OCT studies in dementia patients are scarce with only a few reports (Shen et al., 2013; Choi et al., 2016; Mutlu et al., 2018) suggesting that inner retina layer thicknesses may be a marker for dementia progression (Choi et al., 2016) or development (Mutlu et al., 2018). There is no longitudinal SD-OCT data for FTD to our knowledge. With a group of deeply phenotyped FTD patients followed for about 16 months, our study demonstrates persistent outer retina thinning with no development of inner retina thinning compared to normal controls, and the thinning of the outer retina significantly correlated with the decline in MMSE. The results from this longitudinal study, along with our previously reported cross-sectional study in the same study cohort (Kim et al., 2017), support the outer retina thickness as a potential biomarker for FTD patients.

Although there has been some mild inconsistency in OCT studies of AD patients, the large majority of OCT studies (Coppola et al., 2015; Garcia-Martin et al., 2016), as well as histopathologic data (Hinton et al., 1986; Koronyo et al., 2017), have shown that AD is associated with inner retina thinning (retinal nerve fiber layer and ganglion cell layer) without outer retina abnormalities (Uchida et al., 2018). The exact cause for the inner retina thinning is unclear, but it may be related to Aβ toxicity within the inner retina as opposed to being a reflection of generalized neuronal loss within the central nervous system (Koronyo et al., 2017). Importantly, our longitudinal data of FTD patients did not show any evidence of retinal nerve fiber layer or ganglion cell layer thinning, and we used an image segmentation algorithm that revealed inner retina thinning in other dementia patients (Mutlu et al., 2018). This suggests that inner retina thinning is unlikely to be a late finding for our cohort of patients, and argues that the outer retina thinning we observed is unlikely to have resulted from non-specific neuronal loss. Thus, specific dementias may have specific retinal abnormalities, and our longitudinal data show that the inexpensive, safe, and quick retinal imaging of SD-OCT could help to distinguish FTD from AD.

We found significantly progressive outer retina thinning only in our tauopathy subgroup. This implicates tau pathology in FTD as a cause of the outer retina thinning, although our data does not enable us to comment on the exact mechanism by which a tauopathy affects photoreceptors. While AD also is a tauopathy, tau abnormalities in AD are composed of a mixture of 3R and 4R tau with paired, helical filaments (Irwin et al., 2015). This contrasts with the primarily 3R or 4R alone (not mixed) tauopathy with straight filaments of FTLD-Tau and, along with the Aβ toxicity of AD, may explain differences between retinas of AD and FTLD-Tau. SD-OCT may be most useful as a distinguishing biomarker for FTLD-Tau, and further investigation is needed.

Interestingly, inner retina thinning has been reported in patients with a progranulin mutation and patients with amyotrophic lateral sclerosis, both of which are neurodegenerative diseases associated with a TDP-43 proteinopathy (Ward et al., 2014; Volpe et al., 2015). This also suggests that our finding of outer retina thinning seen in comparing all of our FTD patients to controls may be driven by our tauopathy patients. Further, it indicates that SD-OCT may have potential to distinguish FTLD-Tau from FTLD-TDP. While another report has seen inner retina thinning in a group of 17 FTD patients, patients in this study were not separated into probable tauopathy or TDP-43 subgroups (Ferrari et al., 2017). The inner retina thinning reported in this previous study may have been related to a high proportion of TDP-43 related pathology, but this is unclear without autopsy. Because of the disease heterogeneity of FTD, we believe that SD-OCT studies of these patients should use biomarkers and deep phenotyping whenever possible, as we have done in this study. Our findings remain to be confirmed at autopsy.

Depending on the disease, the inner or outer retina may become edematous (thicken) before later developing atrophic changes (thinning) (Sadigh et al., 2013; Rovere et al., 2015). Longitudinal data are therefore needed to determine if a potential retinal biomarker found in a cross-sectional study remains a significant finding over time. In this study, we found that thin layers did not become thickened or vice versa. Interestingly, we also found significant rates of change in retinal layers not involving photoreceptors. There was a significant rate of OPL thickening for the tauopathy subgroup, and this corresponds with non-significant OPL thickening seen in our cross-sectional data. In contrast to other retina layers, the OPL can be difficult to accurately segment (Oberwahrenbrock et al., 2015). However, two cross-sectional studies of PSP patients compared to normal controls have also shown non-significant thickening of the OPL and thinning of the ONL (Schneider et al., 2014), with significant thinning of the ONL seen in one of the studies (Albrecht et al., 2012). This is important because PSP patients are highly likely to have tau pathology (Litvan et al., 1996; Josephs et al., 2006; Hoglinger et al., 2017). The thickening of the OPL is potentially related to the sprouting of bipolar and horizontal cell dendrites from the OPL into the neighboring ONL as the ONL thins from photoreceptor atrophy (Fariss et al., 2000; Liets et al., 2006; Schneider et al., 2014). We also observed a significant rate of INL thinning compared to controls in both FTD patients and the probable tauopathy subgroup. The INL is the layer that contains the nuclei of bipolar, horizontal, and amacrine cells; it is not part of the inner retina thinning (composed of retinal nerve fiber layer and ganglion cell layer) typically associated with AD. These findings may be related to tau, which has been found within the INL and photoreceptors in the human retina (Leger et al., 2011; Schon et al., 2012).

In our baseline study, we found that the outer retina thickness correlates with the MMSE for FTD patients (Kim et al., 2017). Our longitudinal data shows outer retina thickness correlations with MMSE in several ways, confirming these data. While the baseline SD-OCT measurements did not predict a change of MMSE, there were especially robust findings for the correlation of the change of outer retina thickness with the follow-up MMSE in FTD patients (*p* < 0.001). This suggests that the baseline measurement of the outer retina thickness in these patients was too variable between patients to predict a change in MMSE, but the amount of change over time for this measurement (the rate of change) likely had less variability enabling its correlation with the change in MMSE. Additional studies of correlations with other severity measures specific to FTD are planned.

The strengths of our data are several-fold. Most current biomarker studies in FTD use clinically defined samples only, which limits meaningful interpretations in regards to underlying pathophysiology of this pathologically heterogeneous spectrum of neurodegenerative conditions. We employed rigorous methodology that includes deep endophenotyping of patients with autopsy-validated CSF biomarkers and genetic data to characterize our cohort. While not a complete substitute for autopsy data, AD CSF biomarkers appear to not only differentiate AD from controls, but also from forms of FTLD (Shaw et al., 2009; De Meyer et al., 2010; Irwin et al., 2012, 2013; Lleo et al., 2018). Thus, non-fluent PPA and CBS patients in our cohort are unlikely to have primary AD neuropathology, and the clinical diagnosis of PSP is highly specific for FTLD-Tau (Hoglinger et al., 2017). Further, while non-fluent PPA and CBS can also be associated with underlying FTLD-TDP pathology with *GRN* mutation, sporadic FTLD-TDP with these clinical features are rare (Grossman, 2012). Thus, our combination of CSF, genetic, and clinical criteria enable us to exclude AD and define a probable tauopathy subgroup with confidence. As additional study strengths, we performed eye exams to exclude confounding diseases and used a well-validated SD-OCT image segmentation algorithm. While follow-up studies of dementia patients have inherent challenges, we also had excellent retention for this study. It is unlikely that the missing data from subjects lost to follow-up would have altered our key results as the tauopathy patients that were unable to return because of disease progression probably would have had even more outer retina thinning.

The weaknesses of our study are primarily that the sample size of this longitudinal study is limited. Our findings should be replicated in a larger study. Without autopsy data, it is also possible that some patients in our cohort have mixed pathologies as opposed to “pure” FTLD (Kovacs, 2016; Lleo et al., 2018). Still, our multimodal biomarker approach to deeply phenotype our cohort is an excellent approach as autopsy-confirmed data in FTD is rare. Lastly, while our study subjects were consecutively recruited, the controls had different demographic features. Our data accounted for this by including statistical adjustment for age, sex, and race. Furthermore, our results are completely aligned with our baseline study and pre-specified hypothesis, suggesting the veracity of our findings. Larger studies would enable evaluation of the influence of demographic features such as sex on outer retina thickness measurements.

This is the first longitudinal report of SD-OCT retinal imaging in a group of deeply phenotyped FTD patients. Our findings of persistent outer retina thinning in FTD, and progressive outer retina thinning in the probable tauopathy subgroup implicates a potential tau-related mechanism for outer retina thinning. The correlations with disease severity again provide independent evidence of the significance of these findings. SD-OCT thus has potential as a biomarker for FTD. Future studies are aimed at directly comparing probable tauopathy patients to TDP-43 and AD patients across time, ultimately following patients to autopsy to confirm the molecular pathology.

## Author Contributions

BK, MG, JD, and DI contributed conception and design of the study. BK, MG, DS, SS, WP, SD-P, TA, G-SY, and DI performed acquisition and analysis of data. BK, MG, DS, JD, G-SY, and DI drafted the manuscript and figures.

## Conflict of Interest Statement

The authors declare that the research was conducted in the absence of any commercial or financial relationships that could be construed as a potential conflict of interest.
